# Tirzepatide Versus Semaglutide for Weight Loss in Overweight and Obese Adults: A Systematic Review and Meta-Analysis of Direct Comparative Studies

**DOI:** 10.7759/cureus.86080

**Published:** 2025-06-15

**Authors:** Nazish Munawar, Aakash Mahato, Anurag Rawat, Fahad Shaukat Gill, Daksh Kumar, Susant Katwal, Calvin R Wei, Neelum Ali

**Affiliations:** 1 Internal Medicine, Alberta Health Services, Edmonton, CAN; 2 Internal Medicine, Mirbat Hospital, Ministry of Health, Salalah, OMN; 3 Interventional Cardiology, Himalayan Institute of Medical Sciences, Dehradun, IND; 4 Medicine, Shalamar Medical and Dental College, Lahore, PAK; 5 Internal Medicine, Bahria University Medical and Dental College, Pakistan Navy Ship (PNS) Shifa Hospital, Karachi, PAK; 6 Internal Medicine, Rakhyut Hospital, Ministry of Health, Rakhyut, OMN; 7 Research and Development, Shing Huei Group, Taipei, TWN; 8 Internal Medicine, University of Health Sciences, Lahore, PAK

**Keywords:** meta-analysis, obesity, semaglutide, tirzepatide, weight loss

## Abstract

This systematic review and meta-analysis compared the weight loss efficacy of tirzepatide versus semaglutide in overweight and obese adults through direct comparative studies. We systematically searched PubMed, Excerpta Medica database (EMBASE), Web of Science, and Cochrane Library databases from inception to April 25, 2025, identifying studies that directly compared tirzepatide and semaglutide for weight management. Inclusion criteria encompassed randomized controlled trials and observational studies reporting percentage change in body weight from baseline. Seven studies totaling 28,980 participants were included, comprising five observational studies and two randomized controlled trials with follow-up durations ranging from six to 12 months. Data analysis was performed using Review Manager 5.4.1 (The Cochrane Collaboration, London, UK) with random-effects models. Results demonstrated that tirzepatide was significantly superior to semaglutide in achieving weight reduction. The pooled analysis showed greater weight loss with tirzepatide compared to semaglutide (standardized mean difference: 0.75, 95% CI: 0.52 to 0.92). At six months, tirzepatide achieved significantly greater weight reduction than semaglutide (mean difference: 1.33, 95% CI: 0.58 to 2.08). Additionally, participants receiving tirzepatide had significantly higher odds of achieving at least 10% weight loss compared to those receiving semaglutide (OR: 0.21, 95% CI: 0.06 to 0.78). High heterogeneity was observed across studies (I² > 90%). This meta-analysis provides evidence that tirzepatide, a dual glucose-dependent insulinotropic polypeptide/glucagon-like peptide-1 (GIP/GLP-1) receptor agonist, demonstrates superior weight loss efficacy compared to semaglutide in overweight and obese populations. These findings support tirzepatide as a more effective pharmacological option for weight management, though further long-term head-to-head trials are needed to confirm sustained benefits and safety profiles.

## Introduction and background

Obesity is a growing global health crisis, affecting more than 650 million adults worldwide [1] and contributing significantly to the burden of non-communicable diseases such as type 2 diabetes mellitus (T2DM), cardiovascular disease, and certain cancers [2]. Despite increasing awareness and public health efforts, the prevalence of overweight and obesity continues to rise, highlighting the urgent need for effective and sustainable weight management strategies [3]. Pharmacotherapy has emerged as a valuable adjunct to lifestyle modification for individuals who are overweight or obese, especially those with weight-related comorbidities [4]. Among the available pharmacologic options, glucagon-like peptide-1 receptor agonists (GLP-1 RAs) and dual incretin receptor agonists have demonstrated promising results in promoting clinically meaningful weight loss [5].

Semaglutide, a long-acting GLP-1 RA, has gained significant attention for its substantial weight-reducing effects, particularly in individuals with or without T2DM [6]. Administered once weekly, semaglutide enhances satiety, delays gastric emptying, and reduces food intake, leading to consistent weight loss across multiple randomized controlled trials (RCTs) [7,8]. It is currently approved for chronic weight management in adults with a body mass index (BMI) ≥30 kg/m² or ≥27 kg/m² with at least one weight-related comorbidity [9]. However, the emergence of tirzepatide, a novel dual glucose-dependent insulinotropic polypeptide (GIP) and GLP-1 receptor agonist, has introduced a new therapeutic paradigm [10]. By targeting both GIP and GLP-1 receptors, tirzepatide has demonstrated superior glycemic control and weight loss compared to existing agents, including semaglutide, in recent phase 3 trials [11].

Indirect comparisons have evaluated the efficacy of tirzepatide versus semaglutide in overweight or obese populations, with or without T2DM [12,13]. Preliminary evidence suggests that tirzepatide may induce greater reductions in body weight; however, findings vary across studies in terms of magnitude, safety profile, and population characteristics [12]. Nevertheless, there is a lack of studies that have directly compared semaglutide and tirzepatide in overweight and obese subjects. Recently, studies have been conducted that directly compared semaglutide and tirzepatide in overweight and obese subjects in terms of weight loss effectiveness. Therefore, we are conducting this meta-analysis to compare semaglutide and tirzepatide in overweight and obese subjects.

## Review

Methodology 

We report our methods and results in accordance with the Preferred Reporting Items for Systematic reviews and Meta-Analyses (PRISMA). 

Information Sources and Searches 

We independently searched online databases from the inception of databases to 25 April 2025. Databases used to search relevant articles included PubMed, Excerpta Medica database (EMBASE), Web of Science, and Cochrane Library. Our search strategy included both free-text and medical subject headings (MeSH) terms, utilising the keywords “tirzepatide”, “ly3298176”, “semaglutide”, and “nn9535”. Additionally, we also manually screened included studies and review articles to find any additional studies relevant to the study objectives. Search was performed by two authors. Any disagreement between the two authors was resolved through consensus. 

Eligibility Criteria 

We included observational studies or RCTs that compared the effectiveness of semaglutide and tirzepatide directly and reported the change in weight from baseline (in %). We included studies irrespective of the dose or route. We excluded studies that compared either of these two drugs with a placebo or any other drug or treatment. We excluded meta-analyses, reviews, editorials, and animal studies. 

Study Selection 

Following the removal of duplicate records, the remaining citations underwent an initial screening based on their titles and abstracts to assess relevance to the review objectives. Subsequently, full-text articles of potentially eligible studies were retrieved and evaluated against the predefined inclusion and exclusion criteria. Reasons for exclusion at the full-text stage were meticulously documented to ensure transparency. The screening and selection process was independently conducted by two reviewers. Any discrepancies or disagreements between the reviewers were resolved through discussion; if consensus could not be reached, a third reviewer was consulted to make the final decision. This approach aligns with established best practices for systematic reviews, promoting methodological rigor and minimizing selection bias. 

Data Collection 

Utilizing standardized data extraction templates, we systematically gathered detailed information on study design, participant demographics, and outcome measures. Our primary focus was on quantifying the mean change in body weight from baseline. Secondary outcomes encompassed the proportion of participants achieving a weight reduction of at least 10%. To ensure accuracy and consistency, two reviewers independently performed the data extraction process. Any discrepancies or disagreements encountered were addressed through discussion; if consensus was not achieved, a third reviewer was consulted to resolve the issue. This rigorous approach aligns with established best practices in systematic reviews, promoting methodological rigor and minimizing selection bias. 

Quality Assessment 

Quality assessment of included studies was done independently by two authors using the Newcastle Ottawa Scale and the Cochrane Risk of Bias Assessment tool for observational studies and RCTs. Disagreements between the two authors were resolved through discussion 

Data Analysis 

Data analysis was conducted using Review Manager (RevMan) version 5.4.1 (The Cochrane Collaboration, London, UK). For continuous outcomes, specifically the change in body weight from baseline, standard mean differences (SMDs) between intervention and control groups were calculated, accompanied by 95% confidence intervals (CIs). SMDs were utilized because the included studies reported weight loss outcomes in different units (percentage weight change versus absolute weight change in kilograms), necessitating standardization to enable meaningful pooling of results across studies. Dichotomous outcomes, such as the proportion of participants achieving at least a 10% reduction in body weight, were analyzed using odds ratios (ORs) with corresponding 95% CIs. A p-value of less than 0.05 was considered statistically significant. Heterogeneity among studies was assessed using the I² statistic, with values exceeding 50% indicating substantial heterogeneity. To account for variability across studies, a random-effects model employing the inverse variance method was utilized for pooling effect estimates.

Results 

**Figure 1:**
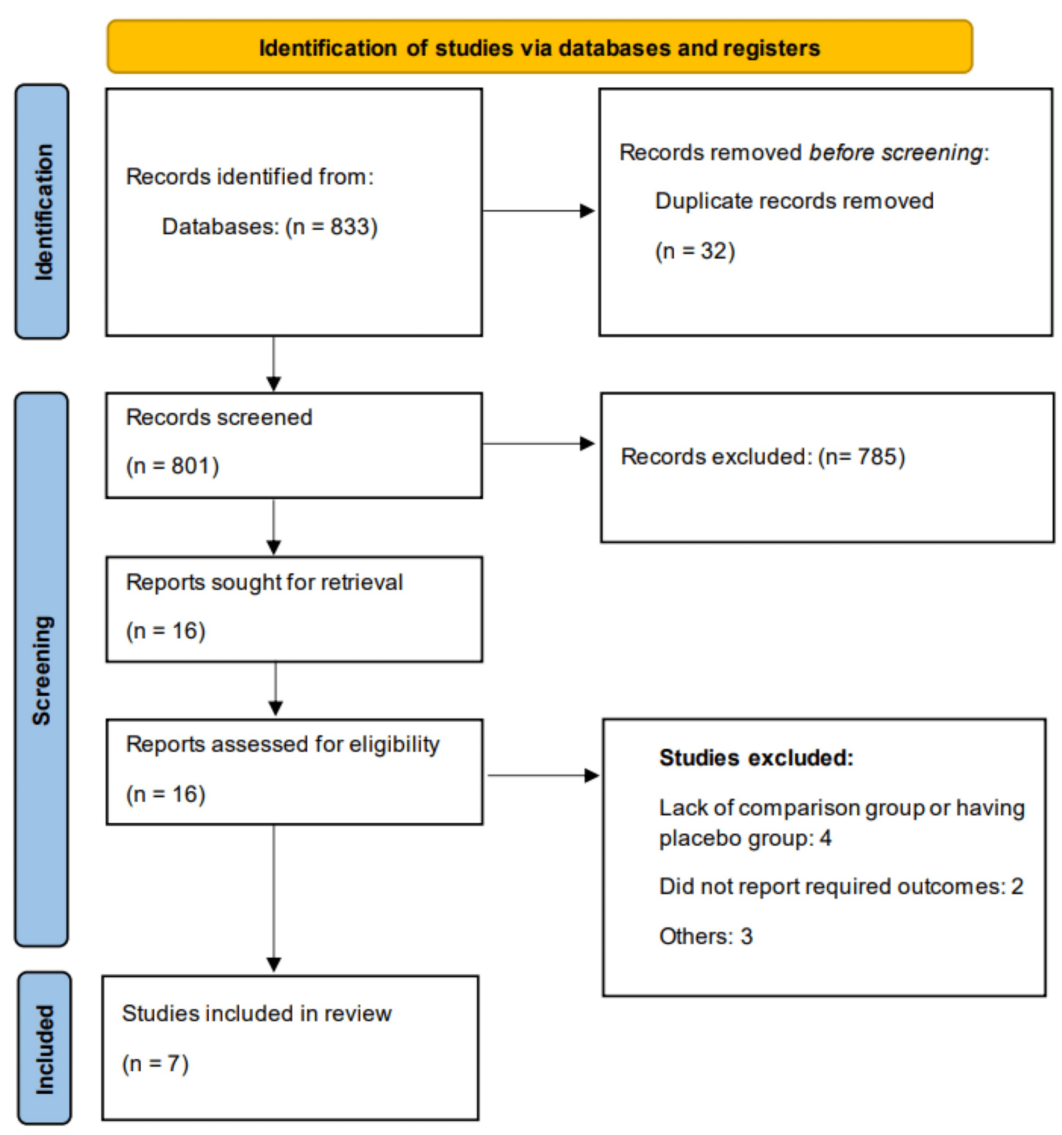
PRISMA flowchart of study selection PRISMA: Preferred Reporting Items for Systematic reviews and Meta-Analyses

Through online database searching, we identified 833 studies. Through initial screening, we found 16 eligible for full-text screening. Ultimately, seven studies were included in this meta-analysis. Figure 1 shows the PRISMA flowchart of study selection. Table 1 shows characteristics of the included studies. Among all included studies, five were observational and two were RCTs. The follow-up duration of included studies ranged from six to 12 months. Table 2 presents the quality assessment of included studies. 

**Table 1:** Characteristics of the included studies NR: not reported

Study ID	Study Design	Follow-up	Groups	Sample Size	Dose	Age (Years)	Male (n)	Diabetes
Anson et al., 2024 [14]	Observational	12 Months	Semaglutide	4223	NR	54	1689	4223
			Tirzepatide	4223		53.9	1689	4223
Frias et al., 2021 [15]	RCT	9 Months	Semaglutide	469	1 mg	56.9	214	469
			Tirzepatide	470	15 mg	55.9	205	470
Gebre et al., 2024 [16]	Observational	9 Months	Semaglutide	50	NR	42	15	50
			Tirzepatide	26		42	12	26
Heise et al., 2023 [17]	RCT	6 Months	Semaglutide	44	1 mg	63.7	34	44
			Tirzepatide	45	15 mg	61.1	31	45
Rodriguez et al., 2024 [18]	Observational	12 Months	Semaglutide	9192	0.5 mg	52	2707	4790
			Tirzepatide	9193	5.0 mg	51.9	2709	4773
Snell-Bergeon et al., 2025 [19]	Observational	12 Months	Semaglutide	50	0.25 mg	42	30	50
			Tirzepatide	50	2.5 mg	39	28	50
Trinh et al., 2025 [20]	Observational	6 Months	Semaglutide	836	NR	52.4	264	480
			Tirzepatide	109		47.5	22	49

**Table 2:** Quality assessment of included observational studies

Author	Selection	Comparability	Outcome	Overall Grade
Anson et al., 2024 [14]	3	2	3	Good
Gebre et al., 2024 [16]	4	2	2	Good
Rodriguez et al., 2024 [18]	4	2	3	Good
Snell-Bergeon et al., 2025 [19]	3	1	3	Good
Trinh et al., 2025 [20]	3	1	2	Good

Change in Weight From Baseline 

**Figure 2:**
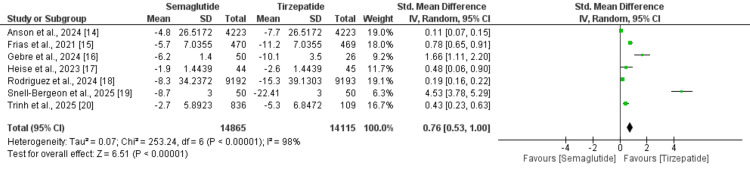
Comparison of the change in weight between the two groups [14-20]

Four studies compared the change in weight between semaglutide and tirzepatide. As shown in Figure 2, the reduction in weight was significantly greater in individuals receiving tirzepatide compared to semaglutide (SMD: 0.76, 95%: 0.53 to 1.00). High heterogeneity was reported among the study results (I-Square: 98%). 

**Figure 3:**
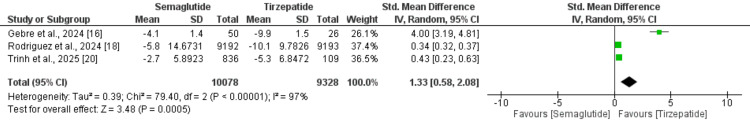
Comparison of change in weight between the two groups (at six months) [16,18,20]

We compared the change in weight between semaglutide and tirzepatide at six months, and the results are presented in Figure 3. As shown in pooled analysis, reduction in weight at six months was significantly greater in individuals receiving tirzepatide compared to semaglutide (SMD: 1.33, 95%: 0.58 to 2.08). High heterogeneity was reported among the study results (I-Square: 97%). 

Proportion of Subjects With at Least 10% Weight Loss 

**Figure 4:**
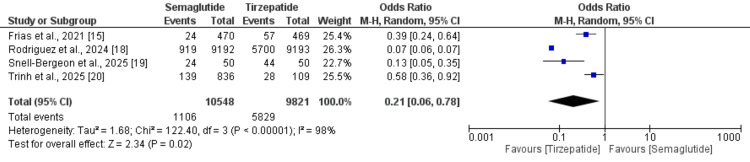
Forest plot comparing the number of subjects with at least 10% weight loss [15,18-20]

Three studies compared the number of subjects with at least 10% weight loss between semaglutide and tirzepatide, and the results are presented in Figure 4. As shown in pooled analysis, the odds of subjects with at least 10% weight loss were significantly lower in subjects receiving semaglutide compared to tirzepatide (OR: 0.21, 95% CI: 0.06 to 0.78). High heterogeneity was reported among the study results (I-Square: 98%).

Sensitivity Analysis of Change in Weight From Baseline

A sensitivity analysis was conducted by systematically removing one study at a time to assess the robustness of the pooled estimate for weight change from baseline, and results are presented in Table 3. The results demonstrated consistent findings across all iterations, with standardized mean differences ranging from 0.47 to 1.13, all favoring tirzepatide over semaglutide. The removal of individual studies did not substantially alter the overall effect size or direction of the association, indicating that no single study disproportionately influenced the pooled results. However, high heterogeneity persisted across all sensitivity analyses (I² = 96-98%), suggesting that between-study variability remained substantial regardless of which study was excluded. These findings support the robustness of our primary conclusion that tirzepatide demonstrates superior weight loss efficacy compared to semaglutide.

**Table 3:** Sensitivity analysis SMD: standardized mean difference; CI: confidence interval

Author	SMD (95% CI)	I-Square
Anson et al., 2024 [14]	1.13 (0.35 to 1.61)	98%
Frias et al., 2021 [15]	0.68 (0.46 to 0.91)	97%
Gebre et al., 2024 [16]	0.64 (0.42 to 0.87)	98%
Heise et al., 2023 [17]	0.79 (0.55 to 1.03)	98%
Rodriguez et al., 2024 [18]	1.13 (0.62 to 1.55)	98%
Snell-Bergeon et al., 2025 [19]	0.47 (0.29 to 0.64)	96%
Trinh et al., 2025 [20]	0.82 (0.57 to 1.07)	98%

Discussion

This meta-analysis evaluated the comparative effectiveness of tirzepatide and semaglutide in inducing weight loss, encompassing a total of 28,980 participants. The primary outcome revealed that tirzepatide was associated with a significantly greater reduction in body weight compared to semaglutide. The corresponding forest plot highlights a spectrum of effect sizes across the included studies, underscoring potential variability stemming from differences in baseline patient characteristics and study designs. These variations may be attributable to differences in trial protocols, including inclusion criteria, intervention durations, and dosing regimens. 

The inclusion of both RCTs and observational studies may have contributed to methodological heterogeneity. While RCTs typically enroll more homogeneous populations under controlled conditions, real-world studies often include broader, more diverse patient groups, potentially influencing outcomes. Additionally, inconsistencies in dose escalation strategies and treatment length across studies likely influenced the extent of weight reduction observed. Despite this heterogeneity, the overall trend across studies consistently favored tirzepatide over semaglutide in terms of weight loss efficacy, although the exact magnitude of benefit appeared context-dependent. Findings by Wen et al. [21] similarly reported superior weight loss outcomes with tirzepatide when compared to semaglutide; however, their analysis was limited to individuals with type 2 diabetes, which may not fully reflect outcomes in broader overweight or obese populations. 

In the direct comparative studies included in this meta-analysis, tirzepatide consistently resulted in greater weight reduction than semaglutide, reinforcing the superior clinical potential of dual GLP-1 and GIP receptor agonism. Semaglutide’s effectiveness in promoting weight loss has been extensively demonstrated in the Semaglutide Unabated Sustainability in Treatment of Type 2 Diabetes (SUSTAIN) trial program, where it significantly outperformed both placebo and various active comparators in terms of body weight reduction (all comparisons p < 0.0001) [22]. Furthermore, semaglutide led to a higher proportion of individuals achieving weight loss thresholds exceeding 5% and 10% (p < 0.0001 for both). The Semaglutide Treatment Effect in People With Obesity (STEP) trial series also reported that a 2.4 mg dose of semaglutide was associated with an average body weight reduction of up to 16% [23]. Tirzepatide, on the other hand, has demonstrated robust weight loss efficacy in its own right. Data from the SURpass-MOUnted Tirzepatide (SURMOUNT) clinical trials have shown reductions in body weight of up to 20.9%, establishing tirzepatide as a potent agent in obesity management [24,25]. However, it is important to recognize that the SUSTAIN, STEP, and SURMOUNT trials assessed each agent in isolation rather than in direct comparisons. As such, the findings from this meta-analysis, which aggregates evidence from head-to-head trials, offer more clinically meaningful insights into the relative performance of tirzepatide versus semaglutide in promoting weight loss. 

The collective evidence from this meta-analysis, alongside existing literature, underscores the substantial weight reduction achieved with both semaglutide and tirzepatide. These pharmacological interventions, when integrated with lifestyle modifications such as dietary adjustments and increased physical activity, can lead to enhanced weight loss outcomes. Notably, both agents exhibit a dose-dependent relationship with clinical efficacy; higher doses are associated with greater improvements in glycemic control and weight reduction. Importantly, these benefits are attained while maintaining tolerable safety profiles, even at elevated dosages [21]. 

In this meta-analysis, a comprehensive assessment of the safety profiles of semaglutide and tirzepatide was not feasible due to limited available data; only two studies reported adverse events for both treatment groups. Nonetheless, existing literature indicates that both agents are associated with gastrointestinal (GI) adverse events, which are typically dose-dependent and occur predominantly during the dose-escalation phase. These GI events are generally transient and classified as mild to moderate in severity. 

For semaglutide, GI adverse events such as nausea, vomiting, and diarrhea have been reported, with incidence rates varying across studies. In the SURPASS trials, semaglutide 1 mg was associated with nausea in 18% of participants, diarrhea in 12%, and vomiting in 8%. These events were typically mild to moderate and occurred during the initial weeks of treatment [16]. Tirzepatide has demonstrated a similar GI adverse event profile. In the SURMOUNT-1 trial, the most common adverse events were GI-related and generally mild to moderate in severity, usually occurring during the dose-escalation period. For those treated with tirzepatide, nausea was reported in 24.6% (5 mg), 33.3% (10 mg), and 31.0% (15 mg) of participants; diarrhea in 18.7% (5 mg), 21.2% (10 mg), and 23.0% (15 mg); vomiting in 8.3% (5 mg), 10.7% (10 mg), and 12.2% (15 mg); and constipation in 16.8% (5 mg), 17.1% (10 mg), and 11.7% (15 mg). Treatment discontinuation rates due to adverse events were 4.3% (5 mg), 7.1% (10 mg), and 6.2% (15 mg), compared to 2.6% in the placebo group [18]. These findings suggest that while both semaglutide and tirzepatide are associated with GI adverse events, these are generally manageable and occur primarily during the dose-escalation period. Further research with standardized reporting of adverse events is necessary to fully elucidate the safety profiles of these agents in diverse populations. 

To advance the understanding of the long-term efficacy and safety of GLP-1 receptor agonists like semaglutide and tirzepatide, future research should prioritize extended-duration studies. These investigations are crucial for assessing the sustainability of weight loss and monitoring potential adverse effects over time. Standardizing baseline characteristics across study populations is essential to minimize confounding variables and enhance the comparability of outcomes. Moreover, conducting additional head-to-head trials will provide clearer insights into the relative effectiveness of different GLP-1 receptor agonists. Given the potential for weight regain following the discontinuation of these medications, it is imperative to explore strategies that support weight maintenance post-treatment. Such strategies may include integrating pharmacotherapy with lifestyle interventions like diet and exercise, which have been shown to enhance and prolong weight loss outcomes. 

Study Limitations 

The findings of this meta-analysis should be interpreted in light of several limitations. Firstly, the number of direct comparative trials between tirzepatide and semaglutide remains limited, restricting the robustness of conclusions regarding their relative efficacy. Future research should focus on conducting long-term, head-to-head studies with varied dosing regimens to better understand the sustained weight loss effects and safety profiles of these agents over extended periods. Secondly, there was notable heterogeneity among the included studies concerning patient demographics, baseline characteristics, and dosing protocols, which may influence the generalizability of the results. Thirdly, inconsistencies in the semaglutide dosages employed across different studies could have impacted the comparative outcomes observed. Lastly, the absence of individual participant-level data precluded the possibility of performing detailed subgroup analyses, limiting insights into how specific patient populations may differentially respond to these treatments. Addressing these limitations in future studies will be crucial for optimizing the clinical application of GLP-1 receptor agonists in weight management.

## Conclusions

This systematic review and meta-analysis highlight the potential of tirzepatide as a superior pharmacological option for weight management compared to semaglutide in overweight and obese adults. The dual GIP/GLP-1 receptor agonism mechanism of tirzepatide may offer enhanced therapeutic benefits over single GLP-1 receptor agonism, suggesting important clinical implications for healthcare providers when selecting optimal interventions, particularly in patients with obesity-related comorbidities. Nonetheless, the observed heterogeneity among studies underscores the need for standardized protocols and more homogeneous patient populations in future research. Long-term head-to-head trials with extended follow-up are essential to evaluate sustained weight loss effects, safety profiles, and optimal dosing strategies. Moreover, cost-effectiveness analyses and real-world studies will be vital for guiding clinical practice and informing healthcare policy regarding the use of these novel agents.
